# Sweet Taste Receptor Serves to Activate Glucose- and Leptin-Responsive Neurons in the Hypothalamic Arcuate Nucleus and Participates in Glucose Responsiveness

**DOI:** 10.3389/fnins.2016.00502

**Published:** 2016-11-08

**Authors:** Daisuke Kohno, Miho Koike, Yuzo Ninomiya, Itaru Kojima, Tadahiro Kitamura, Toshihiko Yada

**Affiliations:** ^1^Advanced Scientific Research Leaders Development Unit, Gunma UniversityMaebashi, Japan; ^2^Metabolic Signal Research Center, Institute for Molecular and Cellular Regulation, Gunma UniversityMaebashi, Japan; ^3^Division of Sensory Physiology, Research and Development Center for Taste and Odor Sensing, Kyushu UniversityFukuoka, Japan; ^4^Monell Chemical Senses CenterPhiladelphia, PA, USA; ^5^Department of Cell Biology, Institute for Molecular and Cellular Regulation, Gunma UniversityMaebashi, Japan; ^6^Division of Integrative Physiology, Department of Physiology, School of Medicine, Jichi Medical UniversityShimotsuke, Japan

**Keywords:** feeding, sweet taste receptor, sucralose, glucose, leptin, POMC

## Abstract

The hypothalamic feeding center plays an important role in energy homeostasis. In the feeding center, whole-body energy signals including hormones and nutrients are sensed, processed, and integrated. As a result, food intake and energy expenditure are regulated. Two types of glucose-sensing neurons exist in the hypothalamic arcuate nucleus (ARC): glucose-excited neurons and glucose-inhibited neurons. While some molecules are known to be related to glucose sensing in the hypothalamus, the mechanisms underlying glucose sensing in the hypothalamus are not fully understood. The sweet taste receptor is a heterodimer of taste type 1 receptor 2 (T1R2) and taste type 1 receptor 3 (T1R3) and senses sweet tastes. T1R2 and T1R3 are distributed in multiple organs including the tongue, pancreas, adipose tissue, and hypothalamus. However, the role of sweet taste receptors in the ARC remains to be clarified. To examine the role of sweet taste receptors in the ARC, cytosolic Ca^2+^ concentration ([Ca^2+^]_i_) in isolated single ARC neurons were measured using Fura-2 fluorescent imaging. An artificial sweetener, sucralose at 10^−5^–10^−2^ M dose dependently increased [Ca^2+^]_i_ in 12–16% of ARC neurons. The sucralose-induced [Ca^2+^]_i_ increase was suppressed by a sweet taste receptor inhibitor, gurmarin. The sucralose-induced [Ca^2+^]_i_ increase was inhibited under an extracellular Ca^2+^-free condition and in the presence of an L-type Ca^2+^ channel blocker, nitrendipine. Sucralose-responding neurons were activated by high-concentration of glucose. This response to glucose was markedly suppressed by gurmarin. More than half of sucralose-responding neurons were activated by leptin but not ghrelin. Percentages of proopiomelanocortin (POMC) neurons among sucralose-responding neurons and sweet taste receptor expressing neurons were low, suggesting that majority of sucralose-responding neurons are non-POMC neurons. These data suggest that sweet taste receptor-mediated cellular activation mainly occurs on non-POMC leptin-responding neurons and contributes to glucose responding. Endogenous sweet molecules including glucose may regulate energy homeostasis through sweet taste receptors on glucose-and leptin-responsive neurons in the ARC.

## Introduction

The feeding center in the hypothalamus plays an important role in energy homeostasis. Neurons in the feeding center are activated or suppressed by the molecules reflecting peripheral energy status, including hormones such as ghrelin, leptin and insulin, and nutrients such as glucose, free fatty acids, and amino acids. These neuronal responses are integrated by intracellular signaling and the brain circuit; as a result, feeding and metabolism are controlled (Williams and Elmquist, 2012). Therefore, nutrient sensing is important for the control of energy balance.

There are two types of glucose-sensing neurons in the hypothalamic arcuate nucleus (ARC; Routh et al., 2014). One is the glucose-excited (or glucose-responsive) neurons, which are activated by high concentrations of glucose and inhibited by low concentrations of glucose. They are thought to be satiety neurons. The other is glucose-inhibited (glucose-sensitive) neurons, which are activated by low concentrations of glucose and inhibited by high concentrations of glucose. Glucose-inhibited neurons are thought to be orexigenic neurons. Regarding the mechanism of glucose-sensing in the hypothalamic neurons, several molecules have been proposed, which include Na^+^, K^+^ ATPase, AMP-activated protein kinase, ATP-sensitive K^+^ channel, and glut2 (Oomura et al., 1974; Ibrahim et al., 2003; Bady et al., 2006; O'Malley et al., 2006; Claret et al., 2007; Routh et al., 2014; Kurita et al., 2015). However, the precise mechanisms underlying glucose-sensing in the hypothalamus are not fully understood.

Glucose-receptor hypothesis was first proposed some decades ago, and recent progress has suggested the sweet taste receptor as the candidate molecule for glucose receptor (Kojima et al., 2015). The sweet taste receptor is a heterodimer of taste type 1 receptor 2 (T1R2) and taste type 1 receptor 3 (T1R3), and this receptor senses multiple sweet taste molecules. In the body, there are variety of sweet taste molecules including carbohydrate, amino acids, polypeptides, proteins, glycosides, and glycerol. T1R2 and T1R3 are distributed throughout multiple organs including the tongue, intestine, pancreas, brain, testis, and lung (Ren et al., 2009; Li, 2013). Recent studies have shown that sweet taste receptors in the pancreatic β cells are necessary for the first phase of glucose response (Nakagawa et al., 2009, 2015). In the brain, the sweet taste receptor is abundantly expressed in the hypothalamus including ARC and the expression level of sweet taste receptor in the hypothalamus is affected by metabolic conditions (Ren et al., 2009; Herrera Moro Chao et al., 2016). However, the role of the sweet taste receptor in the regulation of ARC cellular activities is not understood. Here, we explored the potential contribution of sweet taste receptor to glucose-responses in ARC neurons that could be implicated in energy homeostasis.

## Materials and methods

### Materials

Sucralose, leptin, and nitrendipine were purchased from Sigma (Sigma-Aldrich, St. Louis, MO). ω-Contoxin GIVA and ghrelin were purchased from Peptide Institute (Osaka, Japan). All of the other chemicals were purchased from Wako Pure Chemical Industries (Osaka, Japan).

### Animals

All mouse care and experimental procedures were approved by the Institutional Animal Care and Experimentation Committee at Gunma University. Mice were kept at room temperature (20–24°C) with a 12 h light/dark cycle. All mice used in this study were backcrossed to C57B6J more than eight generations. Regular chow (CLEA Rodent diet CE-2; CLEA Japan, Tokyo, Japan) and water were provided *ad libitum*. POMC-GFP mice were kindly provided by Dr. Jeffrey Friedman (Pinto et al., 2004).

### Preparation of single neurons from ARC

Single neurons were prepared according to procedures reported previously (Kohno et al., 2003, 2007) with slight modifications. Briefly, mice were anesthetized with 10% pentobarbital (10 μl/g) injected intraperitoneally (IP) and decapitated. Their brains were taken out and brain slices containing the entire ARC were prepared. The entire ARC was excised from the left and right sides. The dissected tissues were washed with 10 mM HEPES-buffered Krebs-Ringer bicarbonate buffer (HKRB) containing 1 mM glucose. They were then incubated in HKRB supplemented with 20 U/ml papain (Sigma-Aldrich, P4762), 0.015 mg/ml DNase II (Sigma-Aldrich, D-4138), and 0.75 mg/ml BSA (Sigma-Aldrich, A2153) for 16 min at 36°C. This was followed by gentle mechanical trituration. Then, the cell suspension was centrifuged at 100 × g for 5 min. The pellet was resuspended in HKRB and distributed onto coverslips. The cells were kept in moisture-saturated dishes for up to 4 h at RT.

### Measurements of [Ca^2+^]_i_ in single ARC neurons

Cytosolic Ca^2+^ concentration ([Ca^2+^]_*i*_) was measured by ratiometric fura-2 microfluorometry in combination with digital imaging, as previously reported (Kohno et al., 2003, 2007). Briefly, after incubation with 2 μM fura-2/AM (Dojindo, Kumamoto, Japan, F016) for 45 min, the cells were mounted in a chamber and superfused with HKRB at 1 ml/min at 33°C. Fluorescence images due to excitation at 340 and 380 nm were detected every 10 s with a cooled charge-coupled device camera (ORCA-R2 C10600, Hamamatsu Photonics, Hamamatsu, Japan), and the ratio image was produced by an Aquacosmos (Hamamatsu Photonics). The data were obtained from single cells identified as neurons by previously reported procedures (Kohno et al., 2003, 2007); briefly, they have a relatively large diameter (≥10 μm), and their cell bodies are clear and round on phase-contrast microscopy. Cells with astrocyte-like flat morphology were excluded. The data were obtained from cells that met these criteria for neurons.

### Criteria for [Ca^2+^]_i_ responses and expression of results

Agents were administered in the superfusion solution. When increases in [Ca^2+^]_i_ took place within 5 min after adding agents and their amplitudes were 0.25 or larger, they were considered to have responded. In the studies using inhibitor and blockers, when the amplitude of [Ca^2+^]_i_ responses with drug treatment was 40% or smaller than that of [Ca^2+^]_i_ responses without drugs, inhibition was judged to have occurred. Each experiment was based on data prepared from at least 3 mice. A total of 896 neurons were examined.

### Immunohistochemistry

Mice were anesthetized with 10% pentobarbital (10 ml/kg, IP). Then, mice were perfused transcardially with saline and 10% formalin (Wako, 062-01661). Brains were taken out and post-fixed overnight at 4°C. Then, they were transferred to phosphate-buffered saline (PBS, pH 7.4) containing 20% sucrose. The brains were frozen and kept at −80°C until sectioning. Coronal sections were cut at 25 μm using a cryostat (1:5 series). Sections were collected in, transferred to a cryoprotectant solution, and stored at −30°C.

Double immunostaining of T1R2 and POMC were performed as follows. Sections were rinsed in PBS and then blocked with 3% normal donkey serum (NDS; Abcam, Cambridge, UK, ab7475) diluted in PBS containing 0.25% Triton X-100 for 30 min. Next, sections were incubated in goat anti-T1R2 antibody (Santa Cruz Biotechnology, Dallas, TX, SC-22456, 1:50) and rabbit anti-POMC antibody (Phoenix Pharmaceuticals, H-029-30, 1:500) diluted in blocking solution overnight. After rinsing in PBS, sections were incubated with Alexa 488 donkey-anti-goat IgG (Thermo Fisher Scientific, A-11055, 1:400) and Alexa 594 goat-anti-rabbit IgG (Thermo Fisher Scientific, A-21207, 1:400) diluted in 3% NDS for 40 min.

Double immunostaining of T1R3 and POMC were performed as follows. Sections were rinsed in PBS and then blocked with 1.5% normal goat serum (NGS; Rockland Immunochemicals, Gilbertsville, PA, D204-00-0050) diluted in PBS containing 0.25% Triton X-100 for 30 min. Next, sections were treated with streptavidin solution and biotin solution (Vector laboratories, Burlingame, CA, SP-2002) for 15 min, respectively. They were then incubated in rabbit anti-T1R3 antibody (Abcam, ab65423, 1:1000) diluted in Can Get Signal immunostain solution A (Toyobo, Osaka, Japan, NKB-501) overnight. Sections were rinsed in PBS and incubated with biotinylated goat anti-rabbit IgG (Vector Laboratories, VA-1000, 1:400) for 40 min and incubated with ABC reagent (Vector Laboratories) for 40 min. Sections were rinsed in 0.1 mM Tris-HCl buffer (pH 7.5) containing 0.15 mM NaCl and 0.05% Tween 20, and they were blocked with 0.05% blocking reagent (PerkinElmer, Waltham, MA). They were then treated with biotinyl tyramide (PerkinElmer, NEL700001KT, 1:50) for 5 min. After rinsing in 0.1 mM Tris-HCl buffer (pH 7.5) containing 0.15 mM NaCl and 0.05% Tween 20, sections were incubated with streptavidin-Alexa 488 conjugate (Thermo Fisher Scientific, Waltham, MA, S-11223, 1:500) diluted in blocking reagent for 40 min. After a rinse in PBS, sections were incubated with anti-POMC antibody (1:500) diluted in Can Get Signal immunostain solution A overnight. After rinsing in PBS, sections were incubated with Alexa 594 goat-anti-rabbit IgG (Thermo Fisher Scientific, A-21207, 1:400) diluted in 1.5% NGS for 40 min.

Slices were mounted on slides and coverslipped with mounting medium (Vector Laboratories, H-1200). Fluorescence images were acquired with a BZ-9000 (Keyence, Tokyo, Japan). Confocal florescence images were acquired with a confocal laser-scanning microscope (FV10i, Olympus, Tokyo, Japan).

### Intracerebroventricular (ICV) administration of sucralose and c-Fos and POMC immunohistochemistry

ICV administration was performed as reported previously (Sasaki et al., 2010) with slight modification. Mice were anesthetized with 10% pentobarbital (7 μl/g, ip) and 5% xylazine (10 μg/g, ip) and guide cannula (C315G, Plastics One, Roanoke, VA) was implanted to the right lateral ventricle, and secured to the skull with dental cement (QUICK RESIN, SHOFU, Kyoto, Japan) and adhesion bond (Loctite 454, Henkel, Dusseldorf, Germany). The cannula tip was located 0.2 mm caudal and 1.0 mm right to bregma and 2.5 mm below the skull. More than 1 week after the surgery, mice that had recovered to 90% of their preoperative weight were used for injection. PBS or 0.085 mg sucralose diluted in PBS (0.5 μl) were injected. Thirty min after injection, mice were perfused transcardially with saline and 10% formalin.

Double immunostaining of c-Fos and POMC were performed as follows. Sections were rinsed in PBS, and then treated with 0.3% H_2_O_2_ diluted in PBS for 15 min. Sections were then blocked with 1% BSA and 2% NGS diluted in PBS containing 0.25% Triton X-100 for 30 min. Next, sections were incubated in rabbit anti-c-Fos antiserum (Merck Millipore, PC38, 1:25,000) diluted in blocking solution overnight. After rinsing in PBS, sections were incubated with biotinylated goat anti-rabbit IgG (Vector Laboratories, VA-1000, 1:400) for 40 min and incubated with ABC reagent (Vector Laboratories) for 40 min. Sections were rinsed in PBS and 0.175M sodium acetate buffer (pH 5.6), and color was developed with a nickel-diaminobenzidine solution (10 g/liter nickel ammonium sulfate, 0.2 g/liter DAB, and 0.006% H_2_O_2_ in sodium acetate buffer). After rinsing in PBS, sections were treated with 0.3% H_2_O_2_ diluted in PBS for 15 min. Sections were then blocked with blocking solution. Next, sections were incubated in anti-POMC antibody (1:4000) diluted in blocking solution overnight. After rinsing in PBS, sections were incubated with biotinylated goat anti-rabbit IgG (1:400) for 40 min and incubated with ABC reagent (Vector Laboratories) for 40 min. Sections were rinsed in PBS and 0.1 mM Tris-HCl buffer (pH 7.5) containing 0.15 mM NaCl, and color was developed with a diaminobenzidine solution.

### Statistical analysis

Data are presented as mean ± SEM. Statistical analyses were performed using IBM SPSS Statistics 23. Unpaired Student's *t*-test was used to evaluate differences, with values of *P* < 0.05 considered significant.

## Results

### Sucralose dose-dependently increased [Ca^2+^]_i_ in ARC neurons

**Figure 1:**
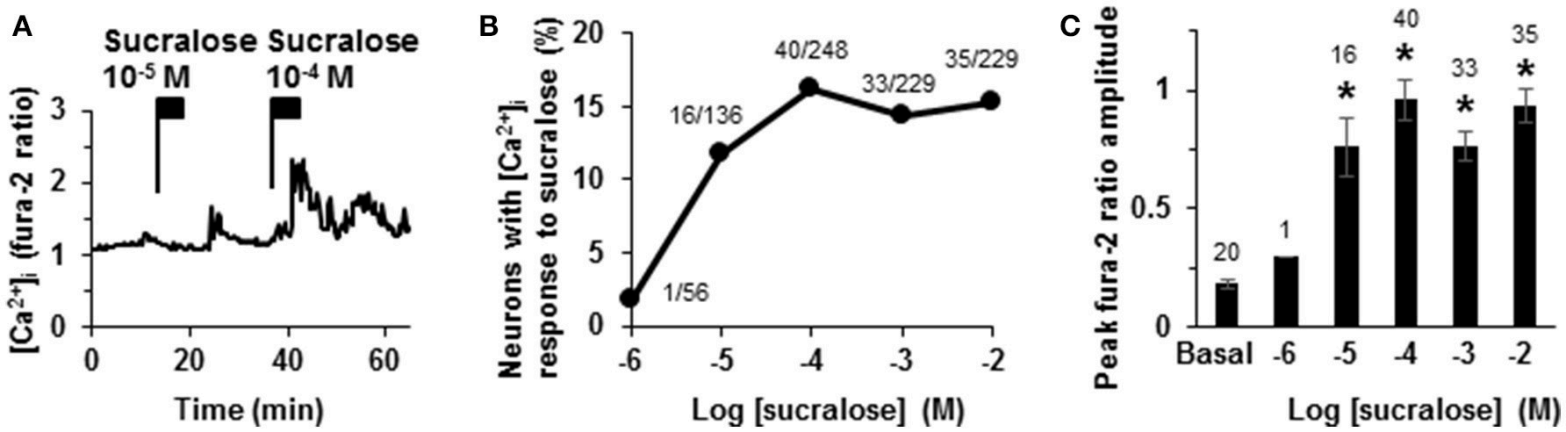
**Increase of cytosolic calcium concentration ([Ca^**2+**^]_**i**_) in ARC neurons after the administration of sucralose. (A)** Sequential addition of sucralose at 10^−5^ and 10^−4^ M increased [Ca^2+^]_i_ in an ARC neuron. **(B)** Response is expressed as the percentage of neurons that exhibited [Ca^2+^]_i_ increases in response to sucralose. The numbers above each point indicate the number of neurons that responded over the number of neurons examined. **(C)** Peak amplitudes of basal oscillation and sucralose-induced [Ca^2+^]_i_ increases are expressed. The numbers above each point indicate the number of neurons examined. Mean ± SEM. Unpaired Student's *t*-test was performed to compare peak amplitudes with basal oscillation peak. ^*^p < 0.001.

To observe the effect of sweet taste molecules on ARC neurons, an artificial sweetener, sucralose, in a concentration range from 10^−6^ to 10^−2^ M was administered to single ARC neurons. Sucralose at 10^−6^ M increased [Ca^2+^]_i_ in 1 of 56 neurons (2%); at 10^−5^ M in 16 of 136 neurons (12%); at 10^−4^ M in 40 of 248 neurons (16%); at 10^−3^ M in 33 of 229 neurons (14%); and at 10^−2^ M in 35 of 229 neurons (15%), showing a concentration-dependent effect (Figures 1A,B). In a neuron presented in Figure 1A, sucralose at 10^−5^ M induced a small [Ca^2+^]_i_ increase, and at 10^−4^ M a sustained [Ca^2+^]_i_ increase with a larger amplitude. Peak fura-2 ratio amplitudes of sucralose-response were concentration dependent. Peak fura-2 ratio amplitudes of response to sucralose at 10^−5^, 10^−4^, 10^−3^, and 10^−2^ M were significantly higher than the peak amplitude of basal fura-2 oscillation level (Figure 1C).

### Sucralose-induced [Ca^2+^]_i_ increases were inhibited by sweet taste receptor inhibitors, under Ca^2+^-free condition, and by L-type calcium channel blocker

**Figure 2:**
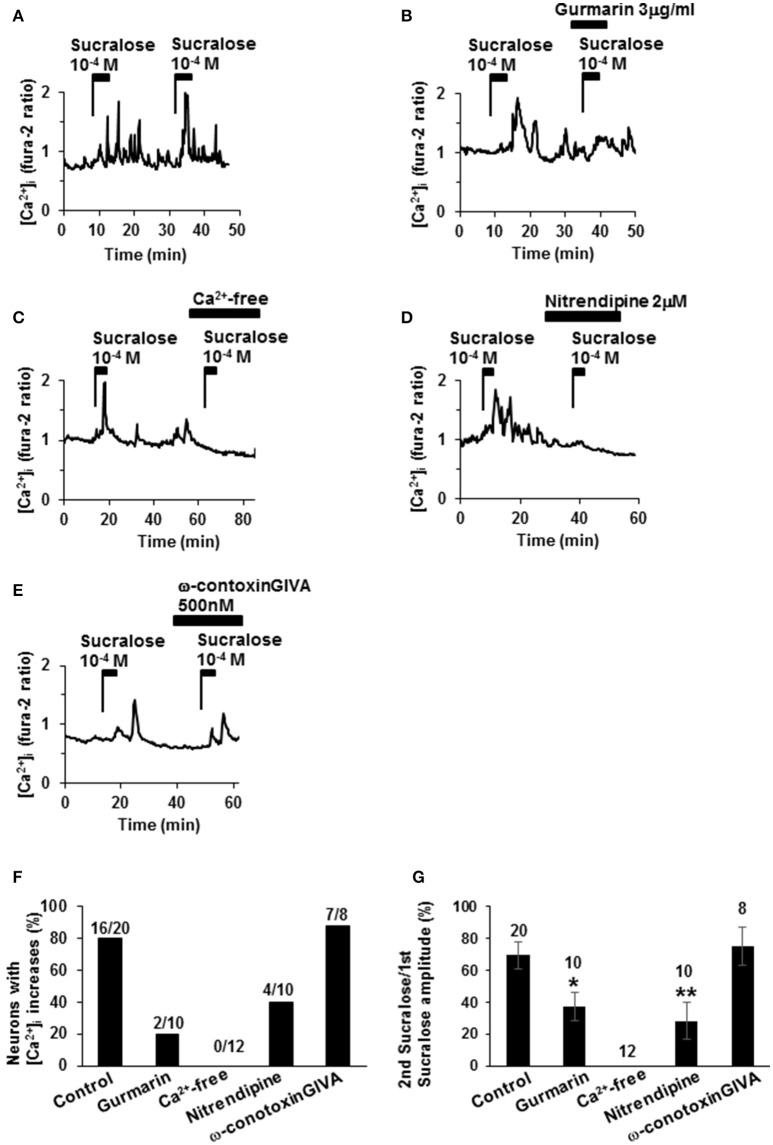
**Mechanisms underlying sucralose-induced [Ca^**2+**^]_**i**_ increases. (A)** Administration of sucralose at 10^−4^ M twice induced repeated [Ca^2+^]_i_ increases in an ARC neuron. **(B)** Sucralose-induced [Ca^2+^]_i_ increases in an ARC neuron were suppressed by gurmarin, an inhibitor of the sweet taste receptor. **(C)** Sucralose-induced [Ca^2+^]_i_increases were abolished under a Ca^2+^-free condition. Sucralose-induced [Ca^2+^]_i_ increases were markedly inhibited by nitrendipine, a blocker of L-type Ca^2+^ channel, at 2 μM **(D)**, while it was unaltered by ω-conotoxin GVIA, a blocker of N-type Ca^2+^ channel, at 500 nM **(E)**. **(F)** Incidence of response under control and test conditions is expressed as the percentage of cells that exhibited [Ca^2+^]_i_ responses to the second addition of sucralose in each cell. The numbers above each bar indicate the number of neurons that responded over the number of neurons examined. **(G)** The amplitude of sucralose-induced [Ca^2+^]_i_ increases under control and test conditions is expressed. The numbers above each point indicate the number of neurons examined. Unpaired Student's *t*-test was performed to compare amplitudes with control. ^*^P < 0.05, ^**^P < 0.01.

The second administration of sucralose induced repeated [Ca^2+^]_i_ increases in 16 of the 20 single-ARC neurons (80%; Figures 2A,F). The second addition of sucralose in each neuron was performed in the presence of drugs or in the absence of Ca^2+^. In the presence of sweet taste receptor inhibitor, gurmarin (Imoto et al., 1991; Shigemura et al., 2008), at 3 μg/ml suppressed sucralose-induced [Ca^2+^]_i_ increase in 8 of 10 neurons (80%; Figures 2B,F). Peak amplitudes of sucralose-induced [Ca^2+^]_i_ increase in the presence of gurmarin were significantly decreased compared with those of control (Figure 2G). These data suggest that the sucralose-induced [Ca^2+^]_i_ increases in ARC neurons are mediated by the sweet taste receptor. Under extracellular Ca^2+^-free condition (added with no Ca^2+^ and 0.1 M EGTA), the [Ca^2+^]_i_ increase in response to sucralose was abolished in all of 12 neurons (100%; Figures 2C,F). The sucralose-induced [Ca^2+^]_i_ increase was suppressed by an L-type Ca^2+^ channel blocker, nitrendipine, at 2 μM in 6 of 10 neurons (60%; Figures 2D,F) and peak amplitudes of sucralose-induced [Ca^2+^]_i_ increase in the presence of nitrendipine were significantly decreased (Figure 2G). In contrast, an N-type Ca^2+^ channel blocker, ω-conotoxin GIVA at 500 nM, failed to affect the sucralose-induced [Ca^2+^]_i_ increase in most [7 of 8 (80%)] of the sucralose-responding neurons (Figures 2E–G). These data suggest that the sucralose-induced [Ca^2+^]_i_ increase in ARC neurons depends on sweet taste receptor and extracellular Ca^2+^ influx, especially through the L-type Ca^2+^ channel.

### Activation in response to high-concentration of glucose is mediated by sweet taste receptor

**Figure 3:**
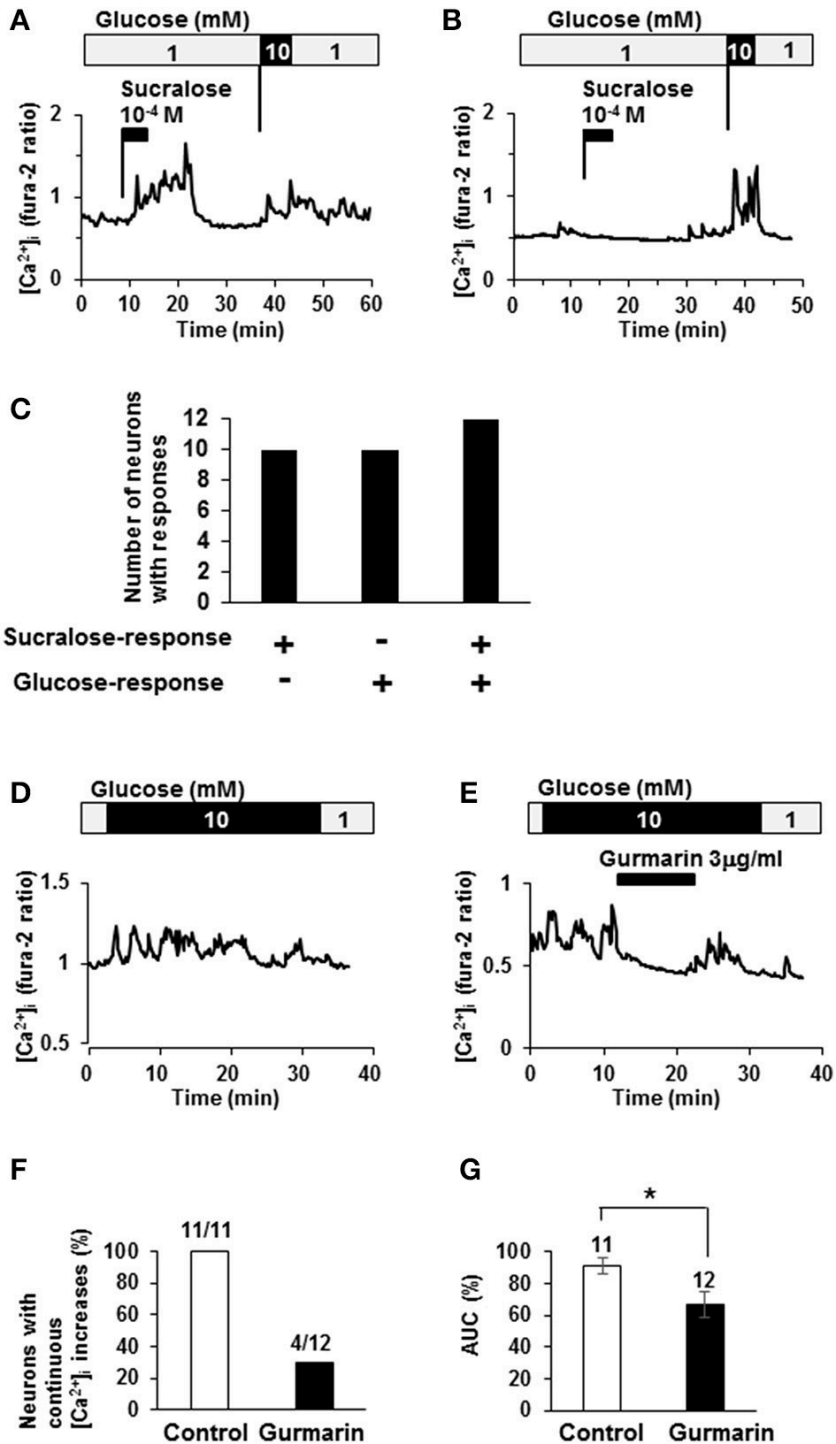
**Relationship between sucralose-response and glucose-sensing. (A)** An ARC neuron exhibited [Ca^2+^]_i_ increases in response to sucralose and raising glucose concentration from 1 to 10 mM. **(B)** An ARC neuron did not exhibit [Ca^2+^]_i_ increase in response to sucralose but did in response to raising glucose concentration from 1 to 10 mM. **(C)** The number of the ARC neurons that responded to sucralose and/or glucose at 10 mM. + responding neurons, – non-responding neurons. **(D)** An ARC neuron exhibited continuous [Ca^2+^]_i_ increase in response to 10 mM glucose. **(E)** In the presence of gurmarin at 3 μg/ml, 10 mM glucose-induced [Ca^2+^]_i_ increase was inhibited. **(F)** Percentage of neurons with continuous [Ca^2+^]_i_ increases in response to 10 mM glucose in the absence or presence of gurmarin. **(G)** Area under the curve (AUC) of fura-2 ratio amplitude in the absence or presence of gurmarin. The numbers above bar indicate the number of neurons examined. ^*^P < 0.05 (Unpaired *t*-test).

The relationship between sucralose-response and glucose response was examined. Among 22 neurons that responded to sucralose at 10^−4^ M, 12 neurons (55%) exhibited [Ca^2+^]_i_ increases in response to elevating the glucose concentration from 1 to 10 mM, the response used to judge the glucose-excited neurons (Figures 3A,C). On the other hand, among 22 neurons that increased [Ca^2+^]_i_ in response to elevating glucose, 12 neurons (55%) exhibited [Ca^2+^]_i_ increases in response to sucralose at 10^−4^ M (Figures 3A–C). Notably, there were no sucralose-responding neurons in glucose-inhibited neurons that decreased [Ca^2+^]_i_ in response to elevating the glucose concentration. These data indicate that more than half of sucralose-responding neurons are glucose-excited neurons, while none of them are glucose-inhibited neurons. These data emphasize the anorexigenic property of sucralose-responding neurons. To explore the contribution of sweet taste receptor to glucose responses, the effect of gurmarin on response to high-glucose was examined. When glucose concentration was increased from 1 to 10 mM, continuous increases of [Ca^2+^]_i_ were observed in 11 out of 11 glucose-excited neurons (100%; Figures 3D,F). The administration of gurmarin at 3 μg/ml suppressed [Ca^2+^]_i_ increases induced by 10 mM glucose in 8 out of 12 neurons (67%; Figures 3E,F). Area under the curve of fura-2 ratio amplitudes in the presence of gurmarin was significantly decreased compared to those in the absence of gurmarin (Figure 3G). These data suggest that excitation induced by high-concentration of glucose is mediated in part by sweet taste receptor.

### Sucralose-responding ARC neurons also responded to leptin but not to ghrelin

**Figure 4:**
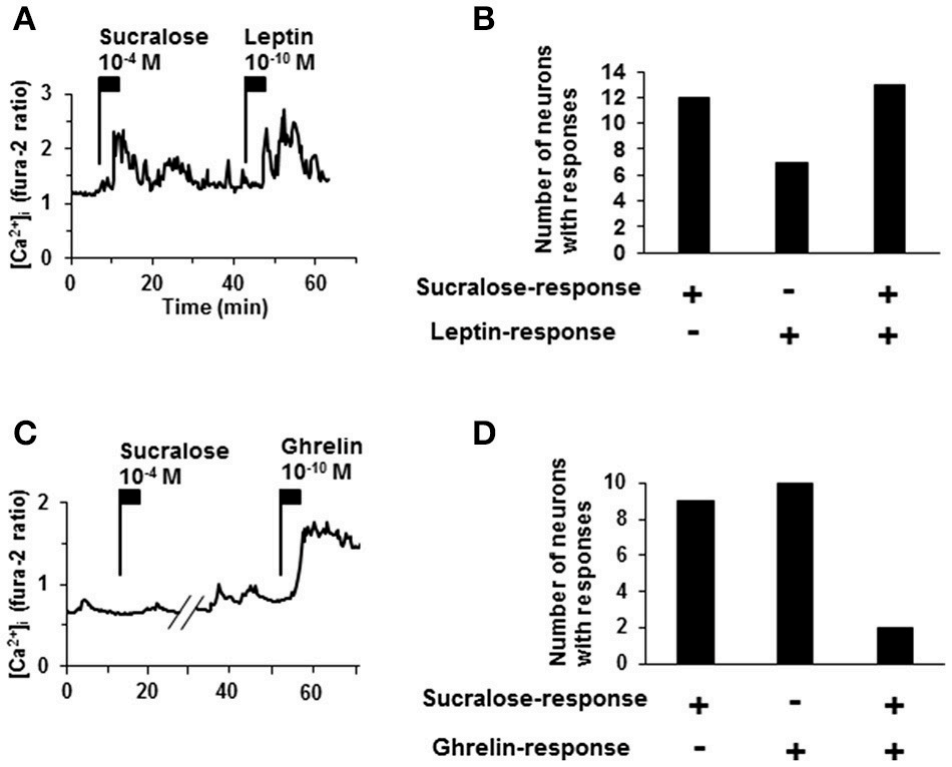
**Relationship of sucralose-response to leptin- and ghrelin-response. (A)** An ARC neuron exhibited [Ca^2+^]_i_ increase in response to both sucralose at 10^−4^ M and leptin at 10^−10^ M. **(B)** The number of responding neurons to sucralose and/or leptin. **(C)** An ARC neurons exhibited [Ca^2+^]_i_ increase in response to ghrelin at 10^−10^ M but not to sucralose at 10^−4^ M. **(D)** The number of the ARC neurons that responded to sucralose and/or ghrelin. + responding neurons, – non-responding neurons.

To further characterize sucralose-responding neurons in ARC, responsiveness to a satiety hormone, leptin, and an orexigenic hormone, ghrelin, (Nakazato et al., 2001; Coppari et al., 2005; Kohno et al., 2007) was examined. Among 25 neurons that responded to sucralose at 10^−4^ M, 13 neurons (52%) exhibited [Ca^2+^]_i_ increases in response to leptin at 10^−10^ M (Figures 4A,B). Among 11 neurons that responded to sucralose at 10^−4^ M, only 2 neurons (18%) exhibited [Ca_2+_]_i_ increases in response to ghrelin at 10^−10^ M (Figures 4C,D). The high overlapping with leptin-responding neurons and low overlapping with ghrelin-responding neurons suggest that sucralose-responding neurons are primarily the neurons implicated in negative energy balance. However, it should be noted that sucralose-responding neurons also include considerable number of non-leptin-responding neurons and ghrelin-responding neurons.

### Sucralose increased [Ca^2+^]_i_ in non-POMC neurons and a few of POMC neurons

**Figure 5:**
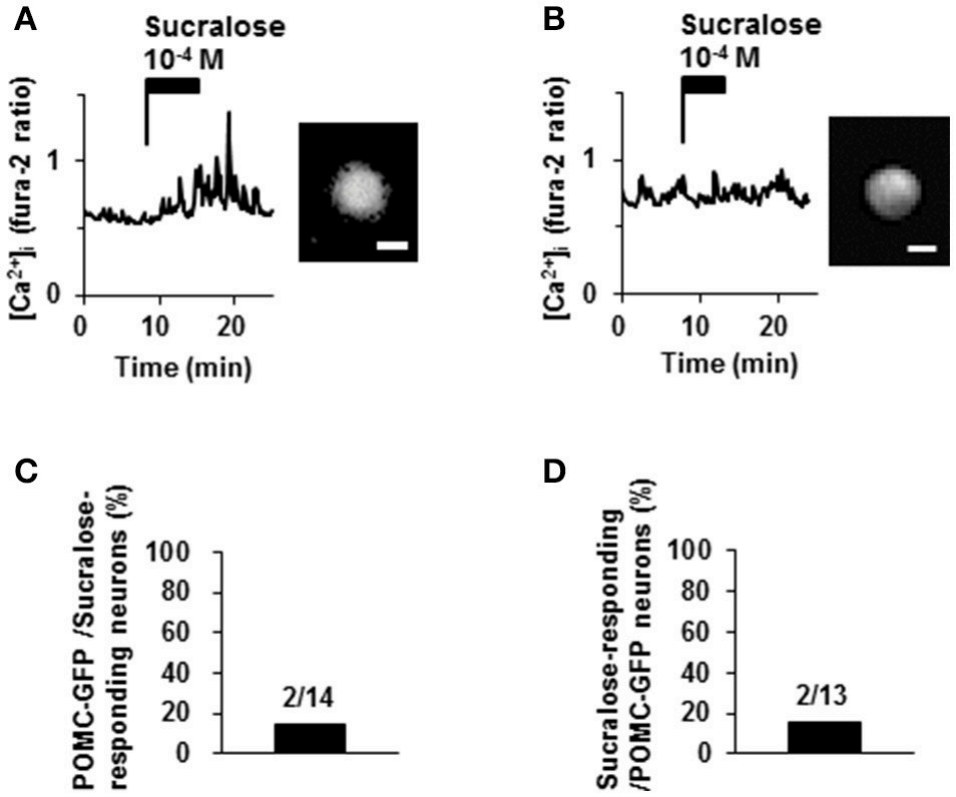
**Effect of sucralose on POMC-GFP neurons. (A)** A neuron with POMC-GFP fluorescent (right) exhibited [Ca^2+^]_i_ increase in response to sucralose (left). **(B)** Another neuron with POMC-GFP fluorescent (right) did not increase [Ca^2+^]_i_ in response to sucralose (left). **(C)** Percentage of POMC-GFP neurons among sucralose-responding neurons in ARC. **(D)** Percentage of sucralose-responding neurons among POMC-GFP neurons. The numbers above bar indicate the number of neurons examined. Scale bar 10 μm.

Proopiomelanocortin (POMC) neurons are major satiety neurons in the ARC. We examined if POMC neurons were large population of sucralose-responding neurons. Among 14 neurons that responded to sucralose at 10^−4^ M, 2 neurons (14%) were POMC-GFP neurons and 12 neurons (86%) were non-POMC neurons (Figures 5A,C). Among 13 POMC-GFP neurons, 2 neurons (15%) exhibited [Ca^2+^]_i_ increase in response to sucralose at 10^−4^ M and 11 neurons (85%) did not exhibit [Ca^2+^]_i_ increase (Figures 5A,B,D). Thus, the majority of sucralose-responding neurons are non-POMC neurons, and only a small part of the POMC neurons responded to sucralose.

### Expression of sweet taste receptor on POMC neurons

**Figure 6:**
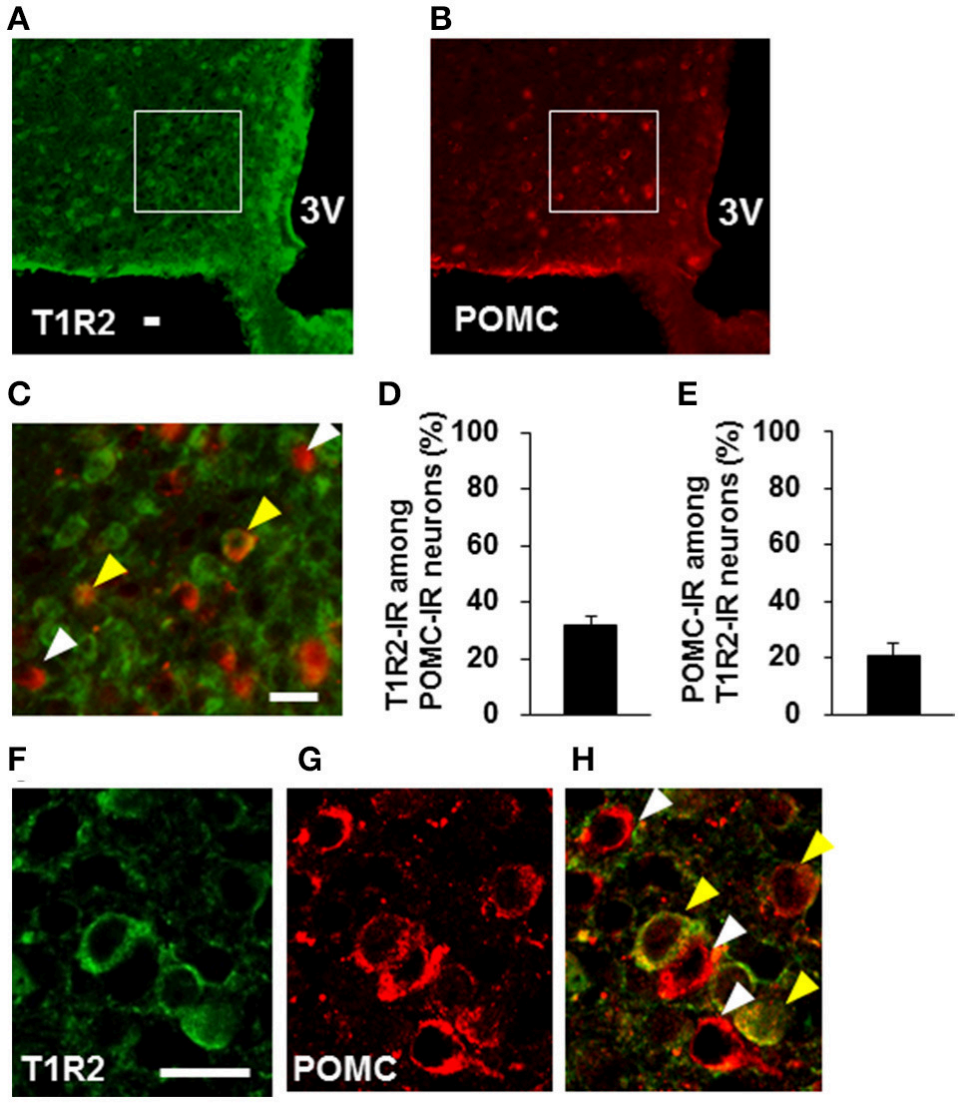
**Distribution of T1R2 on POMC neurons in the ARC**. T1R2 **(A)** and POMC **(B)** immunofluorescent images and their merged image with high magnification **(C)**. **(D)** Percentage of T1R2-imunoreactive (IR) neurons among POMC-IR neurons in the ARC (*n* = 3 brains). **(E)** Percentage of POMC-IR neurons among T1R2-IR neurons in the ARC (*n* = 3 brains). Confocal images of T1R2 **(F)** and POMC **(G)** immunofluorescent and their merged image **(H)**. White arrowhead indicates non-colocalizing POMC-IR neuron, and yellow arrowhead indicates a colocalizing neuron. Scale bar; 20 μm. Graphs show mean ± SEM.

**Figure 7:**
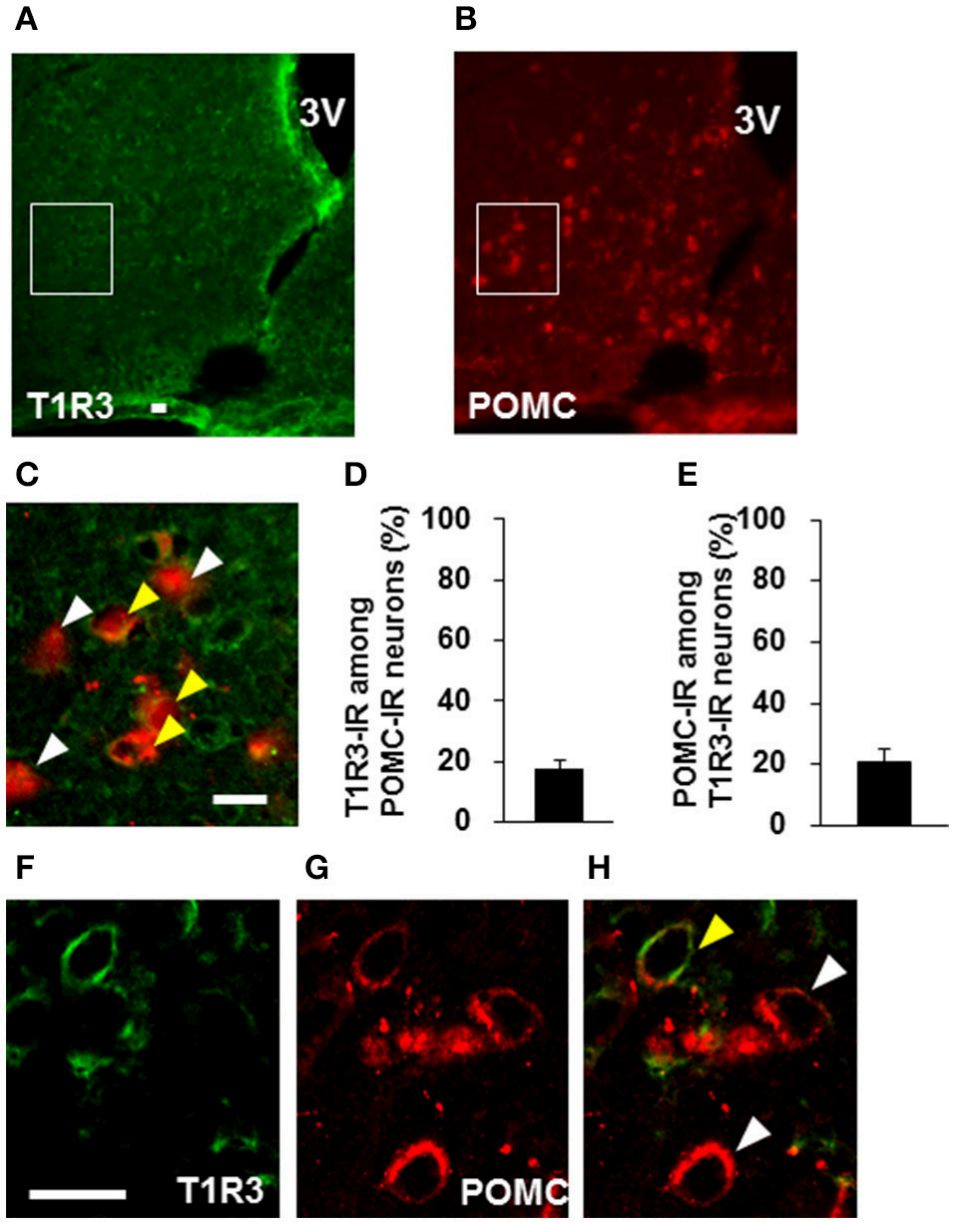
**Distribution of T1R3 on POMC neurons in the ARC**. T1R3 **(A)** and POMC **(B)** immunofluorescent images and their merged image with high-magnification **(C)**. **(D)** Percentage of T1R3-imunoreactive (IR) neurons among POMC-IR neurons in the ARC (*n* = 3 brains). **(E)** Percentage of POMC-IR neurons among T1R3-IR neurons in the ARC (*n* = 3 brains). Confocal images of T1R3 **(F)** and POMC **(G)** immunofluorescent and their merged image **(H)**. White arrowhead indicates non-colocalizing POMC-IR neuron, and yellow arrowhead indicates a colocalizing neuron. Scale bar; 20 μm. Graphs show mean ± SEM.

Colocalization of T1R2 or T1R3 on POMC neurons was examined. T1R2-immunoreactivity was observed on 31.7 ± 3.0 % (*n* = 3 brains) of POMC-immunoreactive (IR) neurons (Figures 6A–D). Among T1R2-IR neurons in the ARC, 20.7 ± 4.7 % (*n* = 3 brains) were POMC-IR neurons (Figures 6A–C,E). T1R3-immunoreactivity was observed on 17.8 ± 2.8 % (*n* = 3 brains) of POMC-IR neurons (Figures 7A–D). Among T1R3-IR neurons in the ARC, 20.5 ± 4.6 % (*n* = 3 brains) were POMC-IR neurons (Figures 7A–C,E). The colocalization was confirmed by confocal microscopy (Figures 6F–H, 7F–H). These data indicate that the sweet taste receptor is expressed on ARC neurons including a part of the POMC neurons, but non-POMC neurons are major property of sweet taste receptor expressing ARC neurons.

### Expression of c-Fos after ICV administration of sucralose

**Figure 8:**
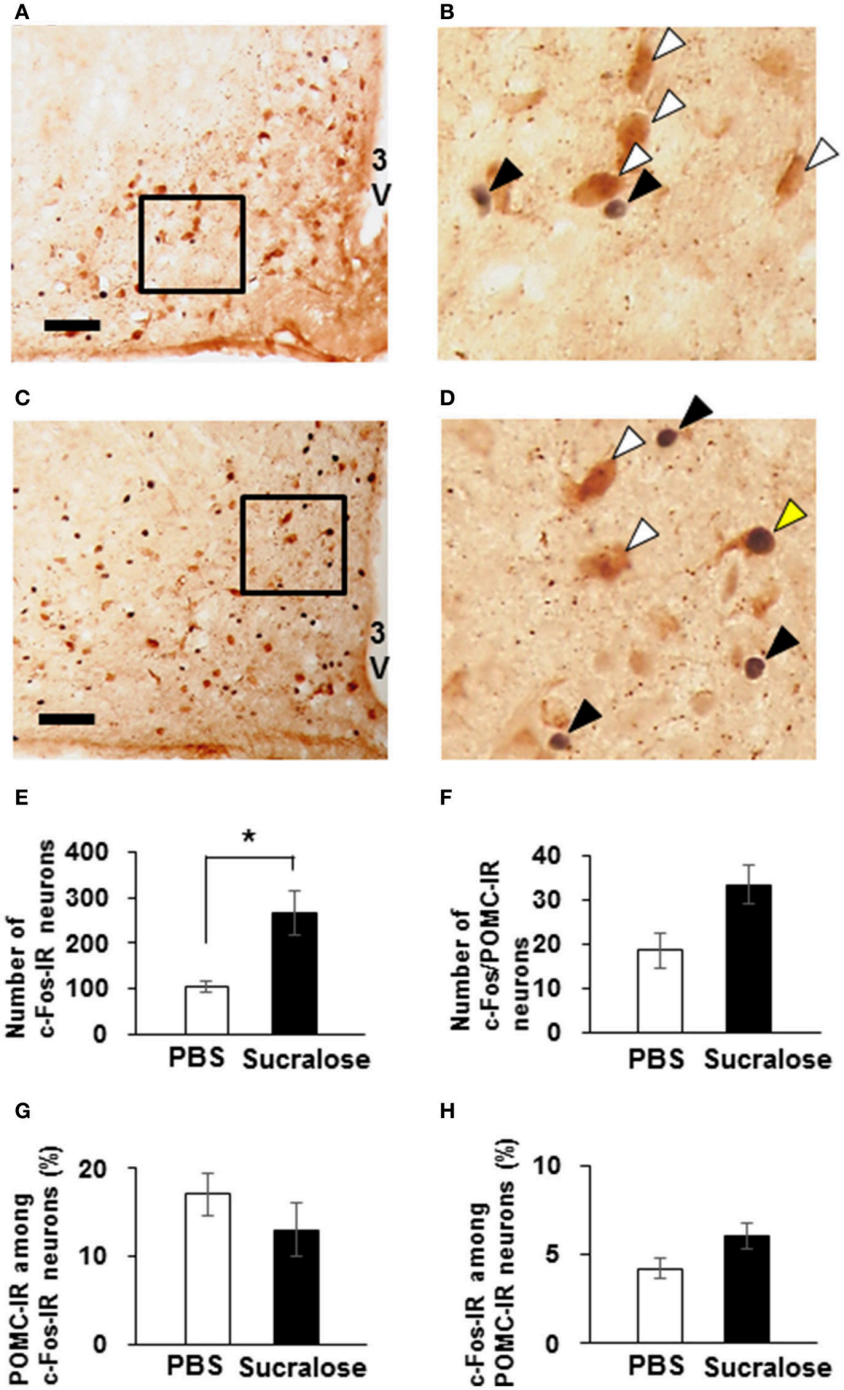
**Expression of c-Fos on POMC neurons after intracerebroventricular (ICV) administration of sucralose**. Mice (*n* = 3) were given a single ICV administration of PBS **(A,B)** or 0.085 mg of sucralose **(C,D)** and killed 30 min later. c-Fos-IR neurons (black) and POMC-IR neurons (brown) were detected in the ARC. Scale bar 100 μm. Black arrowhead, c-Fos-IR neuron, white arrowhead, POMC-IR neurons, yellow arrowhead, neuron immunoreactive to both c-Fos and POMC. **(E)** The number of c-Fos-IR ARC neurons 30 min after ICV administration of PBS or 0.085 mg of sucralose. **(F)** The number of ARC neurons that are immunoreactive to both c-Fos and POMC 30 min after ICV administration of PBS or 0.085 mg of sucralose. **(G)** Percentage of POMC-IR neurons among c-Fos-IR neurons. **(H)** Percentage of c-Fos-IR neurons among POMC-IR neurons. Graphs show mean ± SEM. ^*^P < 0.05 (Unpaired *t*-test).

Using c-Fos protein as a marker of neuronal activation, activation of ARC neurons following ICV administration of 0.085 mg of sucralose was observed. The number of c-Fos-IR ARC neurons were significantly increased in sucralose-administered mice [266.7 ± 50.5 % (*n* = 3 brains)] compared to PBS-administered mice [105.7 ± 12.1 % (*n* = 3 brains); Figures 8A–E]. The number of ARC neurons that were IR to both c-Fos and POMC trended to be increased in sucralose-administered mice [33.3 ± 4.3% (*n* = 3 brains)] compared to PBS-administered mice [18.7 ± 3.9 % (*n* = 3 brains); *P* = 0.07; Figure 8F]. The percentage of POMC-IR neurons among c-Fos-IR neurons (Figure 8G) and the percentage of c-Fos-IR neurons among POMC-IR neurons (Figure 8H) were not altered after sucralose administration. These data indicate that the sucralose in the brain activates ARC neurons, which partly contain POMC neurons.

## Discussion

In this study, we found that an artificial sweetener, sucralose, at 10^−5^–10^−2^ M increases [Ca^2+^]_i_ in ~15% of ARC neurons through the sweet taste receptor-mediated pathway. Sucralose-induced [Ca^2+^]_i_ increases were dependent on the extracellular Ca^2+^ influx at least partly through the L-type Ca^2+^ channel. A large part of sucralose-responding ARC neurons were high-glucose- and leptin-responsive neurons. In addition, response to high concentration of glucose was contributed by sweet taste receptor. Low percentage of POMC neurons were included in sucralose responding neurons, c-Fos expressing neurons, and sweet taste receptor-expressing neurons in the ARC. These findings indicate that sweet taste receptor mediated neuronal activation occurs mainly in high-glucose-and leptin- responsive non-POMC neurons in the ARC, which may contribute to the neuronal activation in response to high-glucose. These mechanisms could be implicated in feeding regulation and energy homeostasis.

There are variety of natural and artificial sweet taste molecules in the body and in food, including carbohydrates, numerous amino acids, metabolites, peptides, glycoside, and artificial sweeteners. Carbohydrates are common natural sweet taste molecules and work as an energy source after being catabolized in each cell. Sucralose is barely catabolized and is thereby non-nutritive. In addition, the sweetness intensity of sucralose is much higher than sucrose (Knight, 1994). To purely observe the effect of the sweet taste molecules and to exclude their nutritive effects, sucralose was used in this study. Indeed, most effects of sucralose were suppressed by the inhibitor of the sweet taste receptor, suggesting that sucralose works as a sweet taste molecule but not as fuel for cellular metabolism.

We found that sucralose at 10^−5^–10^−2^ M induces [Ca^2+^]_i_ increases in ARC neurons. Similarly, it is reported that sucralose at 10^−3^–5 × 10^−2^ M induces [Ca^2+^]_i_ increases in MIN6 cells, a mouse pancreatic β-cell line (Nakagawa et al., 2009). Additionally, sucralose at 2 × 10^−4^–5 × 10^−3^ M induces GLP-1 secretion from NCI-H716 cells, a human enteroendocrine L cell line (Jang et al., 2007). These data support the assumption that sucralose may induce the excitement of cellular activity in a sweet taste receptor-dependent manner. Higher sensitivity of ARC neurons to sucralose could be related to the lower concentration of sweet taste molecules, such as glucose, in the brain compared to the periphery. Glucose concentration in the brain is estimated to be lower than that in blood (Routh et al., 2014).

Sucralose-induced [Ca^2+^]_i_ increases in ARC neurons were dependent on extracellular Ca^2+^ influx, especially through L-type Ca^2+^ channel. Of note, sucralose-induced intracellular signaling could just be a part of the signals that may be induced at the downstream of sweet taste receptors. In pancreatic β-cells and intestinal GLP-1-secreting cells, a variety of sweeteners induce diverse patterns of intracellular signals (Nakagawa et al., 2013; Ohtsu et al., 2014).

In this study, we found sweet taste receptor contributes to the response to high-concentration of glucose. This is similar to role of sweet taste receptor in the pancreatic beta cells where sweet taste receptors serve as glucose receptors (Nakagawa et al., 2009; Hamano et al., 2015; Kojima et al., 2015). RNA expression levels of T1R2 and T1R3 are altered in response to the change of glucose concentration and obesity (Ren et al., 2009; Herrera Moro Chao et al., 2016). Glucose-sensing mediated by sweet taste receptor could be affected by whole body energy status. High-glucose responses are also contributed by some other molecules including AMP-activated protein kinase and ATP-sensitive K^+^ channel (Ibrahim et al., 2003; Claret et al., 2007). In fact, we found that in 33% of glucose-excited neurons, the responses to high-glucose were not altered by the inhibitor of sweet taste receptor, suggesting that high-glucose responses are mediated by sweet taste receptor and other signaling mechanisms. These distinct mechanisms are segregated but could interact with each other. Further studies are required to clarify the precise mechanisms of high-glucose response.

While high-glucose response was suppressed by sweet taste receptor inhibitor in 67% of glucose-excited neurons, sucralose response was observed only in 55% of glucose-responding neurons. This 12% of the difference could be due to the differences between glucose and sucralose as a ligand of sweet taste receptor. The characteristics of sweet taste receptor that can induce multiple downstream signaling cascades depending on ligands (Nakagawa et al., 2013) may have caused the difference between glucose and sucralose in inducing sweet taste receptor-mediated responses.

In this study, 45% of sucralose-responding neurons were not glucose-excited neurons, and also 18% of sucralose-responding neurons were ghrelin-responding neurons. These data suggest that wide variety of neurons could be regulated by sweet taste receptor. Not only glucose but other sweet taste molecules might also be ligands of sweet taste receptor on these neurons. There are several other sweet molecules that can be potential ligands of sweet taste receptor, including amino acids and metabolites, such as glycerol. Comprehensive search of endogenous ligand of sweet taste receptor in the ARC is required for better understanding.

More than half of sucralose-responding neurons also responded to leptin and high concentrations of glucose, but not ghrelin. The concentration of ghrelin in blood rises preprandially while glucose and leptin levels rise post-prandially (Cummings et al., 2001). Ghrelin induces food intake and high-concentration of glucose and leptin induce satiety (Cummings et al., 2001; Nakazato et al., 2001; Routh et al., 2014). These effects are largely mediated by direct actions of ghrelin on neuropeptide Y/AgRP neurons in ARC and by direct excitatory actions of glucose and leptin on satiety neurons including POMC neurons in ARC (Nakazato et al., 2001; Kohno et al., 2003; Coppari et al., 2005). Therefore, the role of ARC neurons in feeding regulation can be suspected by their responsiveness to these key molecules. Sucralose-responding neurons largely responded to both leptin and high-glucose. Inversely, only a minor fraction of sucralose-responding neurons responded to ghrelin. These data implies that the majority of sucralose-responding neurons are those functioning post-prandially or in fed states, which could control feeding, energy metabolism, and glycemia. While POMC neurons are known to be major satiety neurons in the ARC (Williams and Elmquist, 2012), only 14% of sucralose-responding neurons and only 13% of c-Fos-IR neurons were POMC neurons. These results suggest the minor and major contributions of POMC and non-POMC neurons, respectively, to the sweet taste receptor-medicated activation of ARC. This is consistent with previous reports that substantial fraction of glucose-excited neurons in ARC are non-POMC neurons (Wang et al., 2004; Claret et al., 2007; Fioramonti et al., 2007; Parton et al., 2007). While it is well-known that leptin activates POMC neurons in the ARC, leptin's action in the ARC is not limited on POMC neurons. Leptin receptor and leptin-induced immediate signals are observed in ARC neurons including non-POMC neurons (Elias et al., 2000; Lima et al., 2016). There could be unidentified and/or uncharacterized satiety neurons in the ARC. For example, a satiety hormone, nesfatin-1, is localized in ARC neurons other than POMC neurons (Foo et al., 2008). Neurochemical properties of sucralose-responding neurons in the ARC remain to be further characterized.

It is reported that only a small percentage of orally administrated sucralose are absorbed from the intestinal tract in rats, and that most of intravenously administrated sucralose is excreted in urine and feces within 24 h in rats (Sims et al., 2000). It is not yet known if the portion of blood sucralose penetrates the blood-brain barrier (BBB), and the effect of orally ingested sucralose on the hypothalamus is unknown (Schiffman and Rother, 2013). BBB in the ARC is morphologically different from other area (Shaver et al., 1992; Rodríguez et al., 2010; Ciofi, 2011) and is compromised in mice from obese mother (Kim et al., 2016). These characteristics of BBB in the ARC might increase the permeability of sucralose. To clarify the potential side effect of sucralose consumed with foods and to explore the therapeutic application of sucralose, further studies are required.

In this study, we found that the sweet taste receptor is implicated in activation of high-glucose- and leptin- responsive neurons in the ARC, the majority of which are non-POMC neurons. Further, approaches including genetically modified mouse and non-antibody staining could uncover the physiological role and localization of sweet taste receptor in the ARC. Sweet taste receptors may play an important role in coupling systemic factors to neuronal activity and energy homeostasis.

## Author contributions

DK and TY designed research; DK and MK performed experiments and data analysis; YN, IK, and TK contributed to theoretical discussions. DK and TY wrote the paper.

### Conflict of interest statement

The authors declare that the research was conducted in the absence of any commercial or financial relationships that could be construed as a potential conflict of interest.
